# Prognostic Value of Admission Blood Lactate for Predicting 28-Day Mortality in Patients With Septic Shock: A Prospective Pilot Study

**DOI:** 10.7759/cureus.113232

**Published:** 2026-07-23

**Authors:** Hamza El Hamzaoui, Hamza Najout, A Bahouch, Lalla Hasnae Leghlimi, Bouchra Armel, Manal Arfaoui, Abdelkader Benhlima, Maha Louriz, Mustapha Alilou

**Affiliations:** 1 Department of Critical Care and Anesthesiology, Ibn Sina University Hospital, Rabat, MAR; 2 Department of Anesthesia and Critical Care, Mohammed V Military Teaching Hospital, Mohammed V University, Rabat, MAR

**Keywords:** critical care, lactate, mortality, prognosis, septic shock

## Abstract

Background

Septic shock is a leading cause of mortality in intensive care units. Blood lactate is a key biomarker of tissue hypoperfusion; however, the prognostic value of a single initial lactate measurement remains debated. This study aimed to evaluate the predictive value of admission blood lactate levels for 28-day mortality in patients with septic shock.

Methods

A prospective, descriptive, and analytical pilot study was conducted, including 41 patients admitted for septic shock to a multidisciplinary intensive care unit in Rabat, Morocco, over a six-month period. Demographic, clinical, and biological data, including blood lactate levels at admission, were collected. The association between initial lactate and 28-day mortality was analyzed using non-parametric tests, receiver operating characteristic (ROC) curve analysis, and logistic regression.

Results

The mean age of patients was 60.9 years, with a male-to-female ratio of 1.27 (23 men (56.1%) and 18 women (43.9%)). The overall 28-day mortality rate was 56.1% (n = 23). Median admission lactate levels were higher in non-survivors (3.9 mmol/L; n = 23 (56.1%)) than in survivors (2.35 mmol/L; n = 18 (43.9%)), although this difference was not statistically significant (p = 0.231). The ROC curve showed poor discriminative performance (AUC = 0.610; p = 0.227). Logistic regression analysis did not identify admission lactate as an independent predictor of mortality.

Conclusion

Although higher lactate levels were observed in deceased patients, admission blood lactate alone demonstrated limited prognostic value for predicting 28-day mortality in this cohort of septic shock patients. Lactate interpretation should be dynamic and integrated into a comprehensive clinical assessment.

## Introduction

Septic shock remains a major cause of morbidity and mortality in intensive care units worldwide, despite substantial advances in early recognition, antimicrobial therapy, and hemodynamic resuscitation strategies [1,2].

Blood lactate has long been recognized as a key biomarker of tissue hypoperfusion and disease severity in sepsis and septic shock [3,4]. Elevated lactate levels may reflect impaired oxygen delivery, mitochondrial dysfunction, or stress-related metabolic alterations and are therefore widely used for early risk stratification and clinical decision-making in critically ill patients [3]. International guidelines, including the Surviving Sepsis Campaign, recommend lactate measurement as part of the initial evaluation and resuscitation of patients with suspected septic shock [2].

Several studies have demonstrated an association between elevated admission lactate levels and increased mortality in patients with sepsis, both in emergency department and intensive care settings [5,6]. However, the interpretation of lactate values remains complex, as hyperlactatemia may occur in the absence of overt hypoperfusion and may be influenced by multiple pathophysiological mechanisms [7]. As a result, the prognostic value of a single lactate measurement obtained at admission continues to be debated.

Growing evidence suggests that the prognostic relevance of lactate is substantially enhanced when assessed dynamically rather than as a static value [8]. Serial lactate measurements and lactate clearance over time have been shown to better reflect the adequacy of resuscitation and to be more strongly associated with patient outcomes than an isolated baseline value [9,10].

In this context, the present study aimed to evaluate the prognostic value of admission blood lactate levels for predicting 28-day mortality in patients with septic shock admitted to a multidisciplinary intensive care unit.

## Materials and methods

Study design and setting

We conducted a prospective, descriptive, and analytical pilot study over a six-month period, from June to December 2025, in the multidisciplinary intensive care unit of Ibn Sina University Hospital, Rabat, Morocco.

Study population

All consecutive adult patients (≥18 years) admitted to the intensive care unit with a diagnosis of septic shock were eligible for inclusion. Septic shock was defined according to the Sepsis-3 criteria, requiring the presence of sepsis associated with persistent hypotension necessitating vasopressor therapy to maintain a mean arterial pressure (MAP) of ≥65 mmHg despite adequate fluid resuscitation, along with hyperlactatemia [3]. Patients with incomplete clinical or biological data were excluded from the analysis.

Data collection

Demographic characteristics (age, sex), relevant medical history, and clinical data at admission were prospectively collected. Clinical parameters included the qSOFA score, hemodynamic variables, and neurological status. Blood lactate levels were measured at admission as part of routine clinical care. All data were anonymized and recorded in a standardized data collection form.

Outcome measure

The primary outcome of the study was 28-day in-hospital mortality, defined as death from any cause occurring within 28 days following intensive care unit admission.

Statistical analysis

Statistical analyses were performed using MedCalc® statistical software (MedCalc Software Ltd., Ostend, Belgium). Continuous variables were expressed as mean ± standard deviation or median with interquartile range (IQR), as appropriate. Comparisons between survivor and non-survivor groups were conducted using the Mann-Whitney U test for quantitative variables. The discriminatory ability of admission blood lactate to predict 28-day mortality was assessed using receiver operating characteristic (ROC) curve analysis, and the optimal cutoff value was determined using the Youden index. Logistic regression analysis was performed to identify factors associated with mortality. A p-value <0.05 was considered statistically significant.

Given the prospective, observational, and non-interventional design of the study, with data collected exclusively as part of routine clinical care and fully anonymized prior to analysis, the requirement for written informed consent was waived by the ethics committee, and no prior sample size calculation was performed.

## Results

Patient characteristics

A total of 41 patients were included in the study. The mean age was 60.9 ± 13.4 years, with a male predominance (56.1%; n = 23). The most frequent comorbidities were diabetes mellitus (46.3%; n = 19) and active smoking (29.3%; n = 12). The main sources of infection were urogenital (29.3%; n = 12), digestive (26.8%; n = 11), and skin and soft tissue infections (22.0%; n = 9).

At ICU admission, the MAP was 53.8 ± 8.5 mmHg, and 73.0% of patients (n = 30) presented with an altered Glasgow Coma Scale score. The overall 28-day mortality rate was 56.1% (n = 23).

Admission blood lactate levels according to outcome

**Figure 1:**
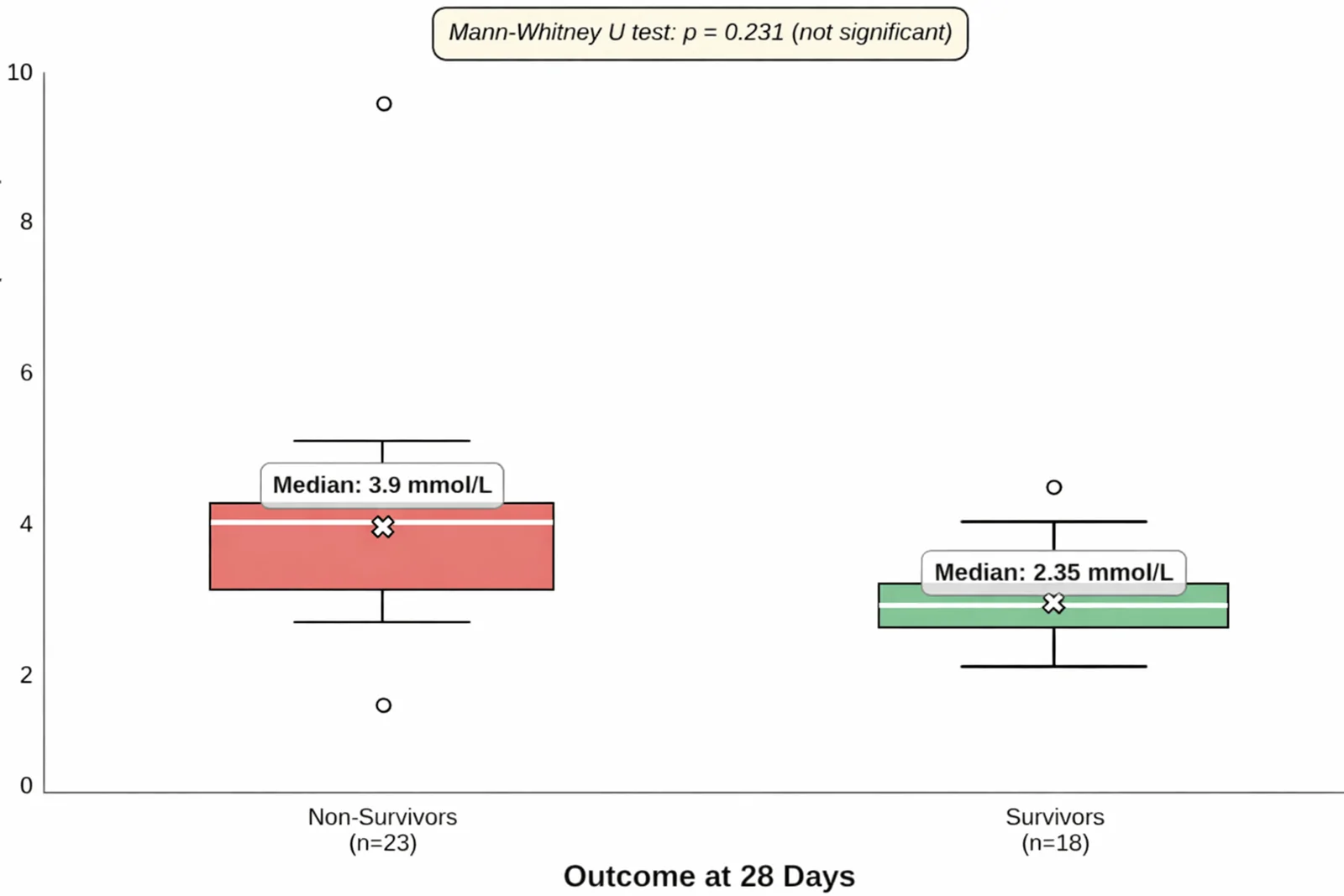
Distribution of Admission Blood Lactate Levels According to 28-Day Outcome in Patients With Septic Shock Box-and-whisker plot showing the distribution of admission blood lactate levels in patients with septic shock according to 28-day outcome. The median lactate level was higher in non-survivors (3.9 mmol/L; interquartile range (IQR): 2.4–5.0) compared with survivors (2.35 mmol/L; IQR: 1.5–4.0). However, the difference between the two groups did not reach statistical significance (Mann–Whitney U test, p = 0.231).

The median blood lactate level at admission in the overall cohort (n = 41) was 3.1 mmol/L (IQR: 1.7-5.0). Patients who died within 28 days (n = 23; 56.1%) had higher admission lactate levels (median 3.9 mmol/L; IQR: 2.4-5.0) compared with survivors (n = 18; 43.9%; median 2.35 mmol/L; IQR: 1.5-4.0). However, this difference did not reach statistical significance (p = 0.231) (Figure 1).

Predictive performance of admission lactate for 28-day mortality

The ROC curve analysis was performed to assess the ability of admission blood lactate levels to predict 28-day mortality. The area under the ROC curve (AUC) was 0.610 (95% confidence interval (CI): 0.445-0.758; p = 0.227), indicating poor discriminative performance.

**Figure 2:**
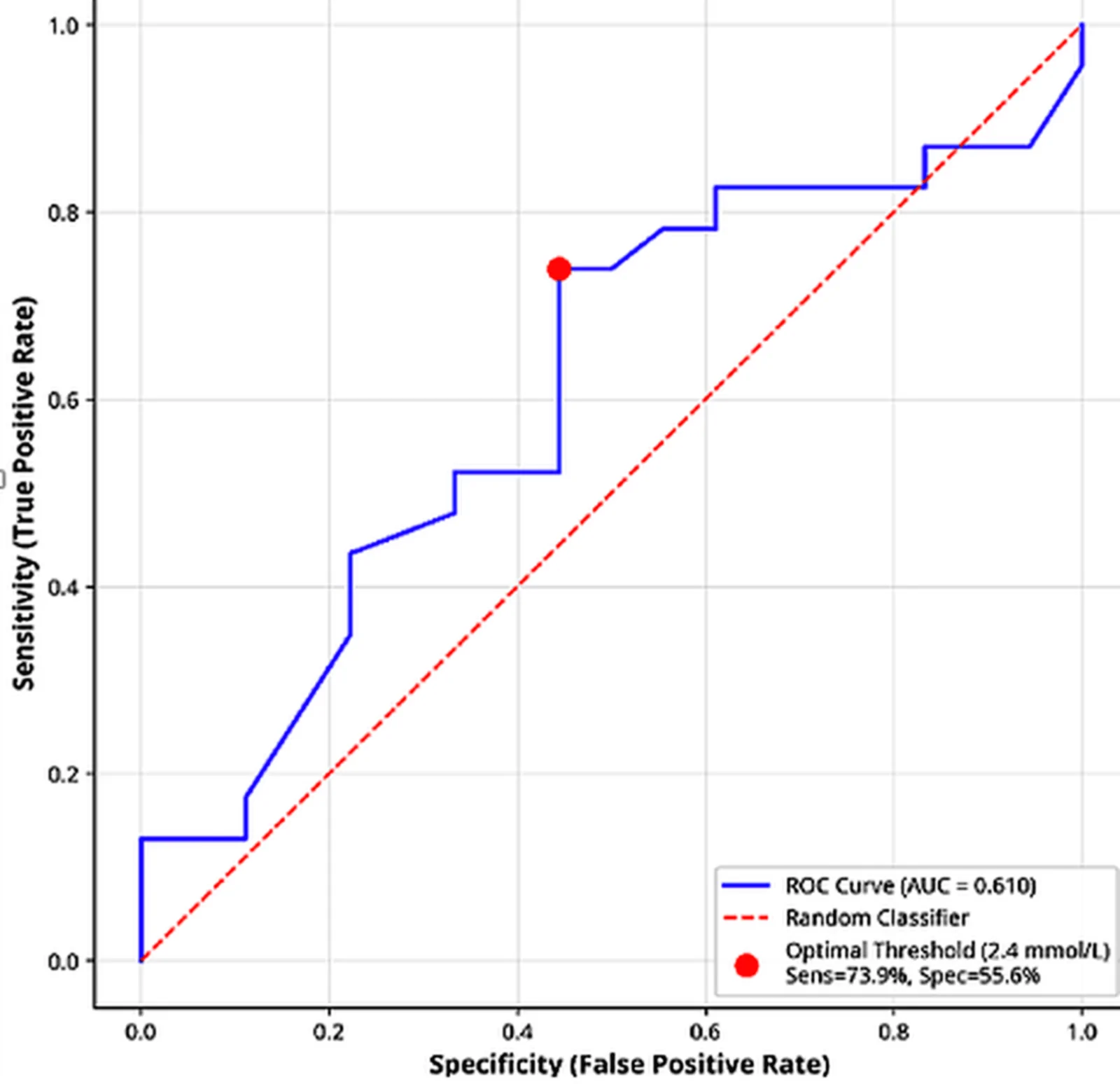
Receiver Operating Characteristic (ROC) Curve of Admission Blood Lactate for Predicting 28-Day Mortality in Patients With Septic Shock Receiver operating characteristic (ROC) curve evaluating the discriminative performance of admission blood lactate levels for predicting 28-day mortality in patients with septic shock. The area under the curve (AUC) was 0.610 (95% confidence interval (CI): 0.445–0.758; p = 0.227). The optimal cutoff value identified using the Youden index was 2.4 mmol/L, corresponding to a sensitivity of 73.9% and a specificity of 55.6%. The dashed diagonal line represents the line of no discrimination.

The optimal cutoff value identified using the Youden index was 2.4 mmol/L, corresponding to a sensitivity of 73.9% (n = 17/23) and a specificity of 55.6% (n = 10/18) (Figure 2).

Logistic regression analysis

In univariate logistic regression analysis, admission blood lactate was not significantly associated with 28-day mortality. Similarly, in multivariate analysis, admission lactate was not identified as an independent predictor of mortality (odds ratio (OR) = 1.42; 95% CI: 0.96-2.11; p = 0.08).

## Discussion

This prospective pilot study evaluated the prognostic value of admission blood lactate levels in patients with septic shock admitted to a multidisciplinary intensive care unit. The observed 28-day mortality rate of 56.1% (n = 23/41) is high but remains consistent with rates reported in international cohorts of septic shock patients, particularly in settings managing severe and complex cases [11,12]. This finding highlights the persistent burden and severity of septic shock despite ongoing advances in critical care management.

The principal finding of this study is the limited prognostic value of a single blood lactate measurement obtained at admission. Although admission lactate levels were higher in non-survivors (n = 23; median 3.9 mmol/L) than in survivors (n = 18; median 2.35 mmol/L), this difference did not reach statistical significance, and the discriminatory performance assessed by ROC curve analysis was poor (AUC = 0.610). These results suggest that initial lactate alone has limited ability to reliably predict short-term mortality in septic shock patients [13].

At first glance, this observation may appear to contrast with the central role attributed to lactate in sepsis management. However, our findings are consistent with an increasing body of literature indicating that while lactate is a valuable marker for early risk stratification and clinical alert, its prognostic relevance is substantially enhanced when evaluated dynamically rather than as a single static measurement [5,14].

The pathophysiology of hyperlactatemia in septic shock is complex and multifactorial. Elevated lactate levels do not exclusively reflect tissue hypoxia due to impaired perfusion (type A lactic acidosis) but may also result from increased aerobic glycolysis driven by catecholamine excess, mitochondrial dysfunction, and reduced hepatic clearance secondary to sepsis-induced organ dysfunction [15]. This complexity likely explains why a single lactate value at admission may inadequately capture the patient’s global physiological status and subsequent clinical trajectory.

Consequently, contemporary clinical practice has shifted from focusing on absolute lactate values to emphasizing lactate kinetics, particularly lactate clearance during the early phases of resuscitation. Several studies have demonstrated that a rapid decrease in lactate levels within the first hours of treatment is strongly associated with improved outcomes and reflects the effectiveness of therapeutic interventions such as fluid resuscitation, vasopressor optimization, and timely antimicrobial therapy [9,14]. The present study, which was designed to assess admission lactate only, did not allow for evaluation of lactate clearance, representing a key limitation.

Despite this limitation, our findings have important clinical implications. They reinforce the concept that elevated admission lactate levels should prompt immediate and aggressive management but should not be used in isolation as a definitive prognostic marker at the initial stage of care. Decisions regarding escalation, limitation, or withdrawal of life-sustaining therapies should not rely solely on a single lactate measurement. Instead, serial lactate monitoring integrated with comprehensive clinical, hemodynamic, and organ function assessments remains the most appropriate approach [14,15].

Several limitations of this study must be acknowledged. The small sample size inherent to its pilot design may have limited statistical power and reduced the ability to detect significant associations. In addition, the single-center nature of the study may limit the generalizability of the results. Future larger, multicenter studies incorporating lactate kinetics and dynamic resuscitation parameters, particularly in low- and middle-income settings, are warranted to better define the prognostic role of lactate in septic shock.

## Conclusions

In this cohort of patients with septic shock, admission blood lactate level was not identified as an independent or robust predictor of 28-day mortality. Although blood lactate remains an essential diagnostic and triage biomarker in the early management of septic shock, its prognostic value should not be based on a single measurement obtained at admission. Instead, lactate interpretation should be dynamic and integrated with comprehensive clinical, hemodynamic, and organ function assessment. Future studies focusing on lactate kinetics, particularly lactate clearance, are warranted to better define its prognostic role and to refine management strategies in our clinical context.
